# Staphylococcus lugdunensis Bacteraemia With Bilateral L4–L5 Facet Joint Septic Arthritis Following Lumbar Radiofrequency Ablation: A Case Report

**DOI:** 10.7759/cureus.104312

**Published:** 2026-02-26

**Authors:** Aqeel Saleem, Zaid Al Hassani, Sanaa Al Ahbabi, Ali Al Hassani, Tariq Hamdan

**Affiliations:** 1 Infectious Disease, Sheikh Tahnoon Medical City, Al Ain, ARE; 2 General Medicine, Sheikh Tahnoon Medical City, Al Ain, ARE; 3 Internal Medicine, Sheikh Tahnoon Medical City, Al Ain, ARE

**Keywords:** bacteraemia, cauda equina symptoms, facet joint septic arthritis, lumbar spine, paraspinal collection, radiofrequency ablation, spinal infection, staphylococcus lugdunensis

## Abstract

Staphylococcus lugdunensis is a virulent coagulase-negative Staphylococcus that can cause invasive bloodstream and deep-seated infection, behaving more like Staphylococcus aureus than commensal skin flora. A 73-year-old patient with chronic low back pain underwent bilateral lumbar medial branch and sacroiliac joint radiofrequency ablation. Over seven weeks, they developed progressively worsening low back pain with bilateral radicular symptoms, saddle anaesthesia, constipation, and acute urinary retention, without fever. C-reactive protein was markedly elevated with a normal white cell count and low procalcitonin. Magnetic resonance imaging demonstrated bilateral L4-L5 facet joint septic arthritis with a small paraspinal collection, without discitis, vertebral osteomyelitis, epidural abscess, or cauda equina compression. Admission blood cultures and computed tomography-guided L4-L5 facet aspiration both yielded S. lugdunensis, confirming bacteraemic bilateral facet joint septic arthritis. Empirical intravenous therapy was initiated and streamlined to intravenous flucloxacillin after susceptibility results and documented microbiological clearance; four weeks of intravenous therapy were completed from the first negative blood culture, followed by prolonged oral step-down therapy. The patient improved clinically, with declining inflammatory markers, radiological interval improvement, and approximately 80% pain reduction without recurrent neurological deficits. Progressive post-procedural pain with neurological red flags after lumbar radiofrequency ablation warrants urgent evaluation for deep spinal infection, even in the absence of fever, and S. lugdunensis bacteraemia should prompt systematic assessment for invasive foci and prolonged antistaphylococcal therapy. This case highlights the need to investigate progressive post-procedural pain with neurological red flags for deep spinal infection, even in the absence of fever, and to treat S. lugdunensis bacteraemia as a true pathogen requiring evaluation for invasive foci and prolonged antistaphylococcal therapy.

## Introduction

Staphylococcus lugdunensis is a coagulase-negative Staphylococcus with virulence that more closely resembles Staphylococcus aureus than other commensal staphylococci [1,2]. It is now recognised as a clinically significant pathogen capable of causing bacteraemia, destructive native valve endocarditis, and invasive bone and joint infection [1-3]. Accordingly, isolation from blood cultures should be treated as clinically meaningful rather than dismissed as contamination, particularly as clumping factor production may lead to misidentification as S. aureus in routine laboratory workflows [1,2]. Contemporary epidemiological data further support its invasive potential across diverse infection syndromes, with emerging oxacillin resistance reported in a minority of isolates [4].

Lumbar radiofrequency ablation is an established intervention for facet-mediated low back pain and is generally considered low risk [5,6]. Most adverse events are minor and self-limiting; however, deep complications such as paraspinal infection and septic arthritis have been reported, albeit infrequently [6,7]. The rarity of severe infection may contribute to diagnostic anchoring on benign post-procedural pain, particularly when systemic features are absent.

Septic arthritis of the spinal facet joint is uncommon and typically involves the lumbar spine, presenting with non-specific back pain and variably with fever [8,9]. Reported recognition has increased with wider use of magnetic resonance imaging (MRI), which is central for defining disease extent, identifying collections, and excluding competing spinal emergencies [8]. In addition, facet joint infection may extend into adjacent paraspinal tissues and, less commonly, the epidural space [8]. Although S. aureus remains the most frequently identified organism, other pathogens, including coagulase-negative staphylococci, are recognised causes [8].

We report a rare case of Staphylococcus lugdunensis bacteraemia with bilateral L4-L5 facet joint septic arthritis and a small paraspinal collection presenting seven weeks after lumbar radiofrequency ablation. We discuss the diagnostic challenges of progressive post-procedural pain without fever, the significance of neurological and autonomic red flags, and key management considerations, including MRI-based evaluation, microbiological confirmation, and prolonged culture-directed antimicrobial therapy.

## Case presentation

A 73-year-old patient with hypertension, dyslipidaemia, benign prostatic hyperplasia, vitamin D deficiency, and chronic low back pain presented with progressive back pain and neurological red-flag symptoms following spinal pain interventions. The patient lived independently and reported good adherence to regular medications (antihypertensives, vitamin D supplementation, and benign prostatic hyperplasia therapy).

Seven weeks before admission, the patient underwent bilateral lumbar medial branch radiofrequency ablation (L2-S1), bilateral sacroiliac joint ablation, and ultrasound-guided trigger-point injections for chronic pain. Five days after the procedure, the patient re-presented to the emergency department with worsening low back pain but no documented focal neurological deficit and was discharged following symptomatic management. Over the subsequent six to seven weeks, pain progressively worsened and became bilateral with radicular symptoms. Shortly before admission, the patient developed saddle anaesthesia, constipation, and acute urinary retention. There was no history of fever.

On admission, the patient was afebrile and haemodynamically stable. Body mass index was 26.13 kg/m². Examination demonstrated severe low back pain with markedly pain-limited spinal movement. Cardiovascular examination was unremarkable, with normal heart sounds and no murmur detected. There were no documented peripheral stigmata of infective endocarditis on examination. A focused neurological examination documented severe pain-limited assessment but no objective motor deficit, with preserved deep tendon reflexes and no documented abnormality of perianal sensation or tone at presentation despite reported saddle anaesthesia and urinary retention. Given the combination of saddle anaesthesia and urinary retention, urgent evaluation for cauda equina syndrome and other spinal emergencies was undertaken. Transthoracic echocardiography showed normal biventricular size and systolic function, no significant valvular abnormality, and no vegetation. The clinical timeline is summarised in Table 1.

**Table 1:** Timeline of key clinical events

Relative time	Clinical event
Day 0	Radiofrequency ablation of bilateral lumbar medial branch nerves (L2 to S1), radiofrequency ablation of bilateral sacroiliac joints, and ultrasound-guided trigger point injections to the neck and shoulder girdle muscles
Day 5	Returned to the emergency department with worsening low back pain; no focal neurological deficit documented; discharged after symptomatic treatment
Admission (hospital day 1)	Afebrile and haemodynamically stable; severe back pain with pain-limited spinal movement; red-flag neurological symptoms prompting urgent evaluation
Weeks 6 to 7	Progressive severe low back pain with bilateral radicular symptoms and sensory complaints; developed saddle anaesthesia, constipation, and acute urinary retention; denied fever

Investigations

Laboratory testing demonstrated a markedly elevated C-reactive protein (114.7 mg/L) with a normal white cell count (8.3-8.8 × 10⁹/L) and low procalcitonin (0.10 ng/mL), supporting a focal inflammatory or infective process in the absence of systemic sepsis. Additional findings included microcytic anaemia and thrombocytosis; renal and hepatic indices were otherwise within reference ranges, apart from mild hypoalbuminaemia. Key laboratory and microbiological results are summarised in Table 2. Admission blood cultures became positive within 24 hours and yielded Staphylococcus lugdunensis; serial cultures documented microbiological clearance with the first negative blood culture on hospital day six (Table 2). The antimicrobial susceptibility pattern of the isolate is presented separately in Table 3 and supported streamlining to targeted beta-lactam therapy.

**Table 2:** Key laboratory and microbiological investigations on admission and during inpatient monitoring

Domain	Test / specimen	Result	Reference range / comment
Inflammatory markers	C-reactive protein	114.7 mg/L	0–5 mg/L
	Procalcitonin	0.10 ng/mL	<0.50 ng/mL
Haematology	White blood cell count	8.3–8.8 × 10⁹/L	4.0–11.0 × 10⁹/L
	Haemoglobin	90–93 g/L	130–170 g/L
	Mean corpuscular volume	74.9–76.4 fL	80–96 fL
	Platelets	443–485 × 10⁹/L	150–400 × 10⁹/L
Renal profile	Sodium	136 mmol/L	135–145 mmol/L
	Potassium	4.9 mmol/L	3.5–5.1 mmol/L
	Chloride	104 mmol/L	98–107 mmol/L
	Bicarbonate	25 mmol/L	22–29 mmol/L
	Urea	4.9–7.6 mmol/L	2.5–7.8 mmol/L
	Creatinine	81.8–85.8 μmol/L	62–106 μmol/L
	Estimated glomerular filtration rate	77–82 mL/min/1.73 m²	≥60 mL/min/1.73 m²
Liver profile	Aspartate aminotransferase	16 IU/L	0–40 IU/L
	Alanine aminotransferase	8 IU/L	0–41 IU/L
	Alkaline phosphatase	64 IU/L	40–129 IU/L
	Total bilirubin	11.7 μmol/L	3–21 μmol/L
	Albumin	33 g/L	35–50 g/L
Microbiology	Blood culture (admission)	Positive within 24 hours	Gram-positive cocci in clusters; *Staphylococcus lugdunensis* isolated
	Blood cultures (serial monitoring)	First negative on hospital day 6	Microbiological clearance documented

**Table 3:** Antimicrobial susceptibility profile of the Staphylococcus lugdunensis isolate Abbreviations: MIC = minimum inhibitory concentration.

Antibiotic / test	MIC interpretation	MIC value (where reported)
Benzylpenicillin	Susceptible (S)	≤0.03 to 0.06
Cefoxitin screen	Negative	Negative
Oxacillin	Susceptible (S)	0.5
Clindamycin	Susceptible (S)	≤0.25
Inducible clindamycin resistance (D-test)	Negative	Negative
Erythromycin	Susceptible (S)	≤0.25
Gentamicin	Susceptible (S)	≤0.5
Linezolid	Susceptible (S)	1.0
Teicoplanin	Susceptible (S)	≤0.5
Tetracycline	Susceptible (S)	≤1.0
Trimethoprim/sulfamethoxazole	Susceptible (S)	≤10.0
Vancomycin	Susceptible (S)	≤0.5

**Figure 1:**
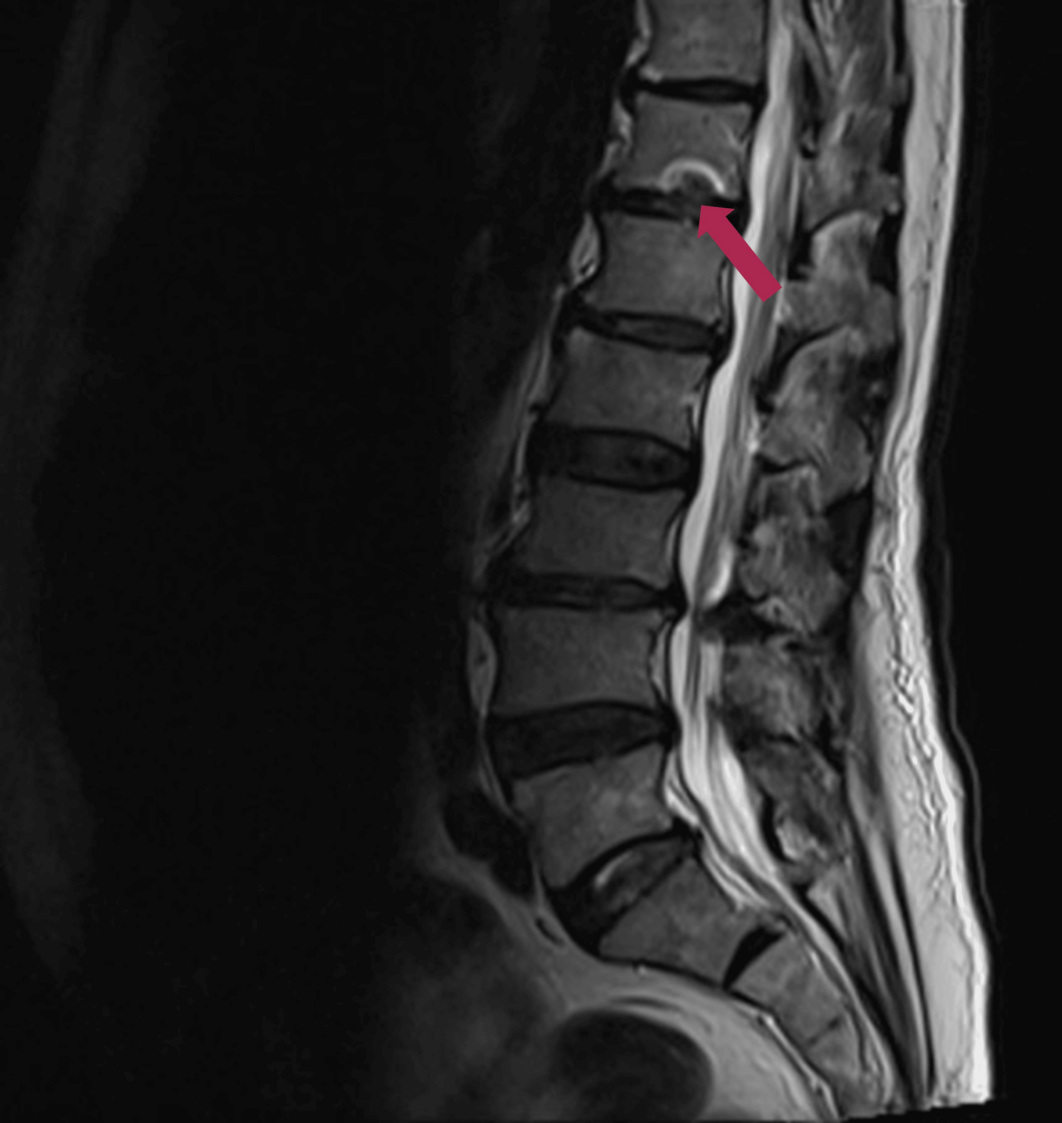
MRI of the lumbar spine demonstrating L4 to L5 facet joint septic arthritis with a small paraspinal collection Bilateral L4 to L5 facet joint septic arthritis with adjacent paraspinal collection (11.5 × 11.2 mm) and milder L3 to L4 inflammatory changes. No discitis, epidural abscess, or cauda equina compression.

MRI of the lumbar spine demonstrated bilateral L4-L5 facet joint septic arthritis with a small adjacent paraspinal collection (11.5 × 11.2 mm), with milder inflammatory change at L3-L4. There was no evidence of discitis, vertebral osteomyelitis, epidural abscess, or cauda equina compression (Figure 1).

In view of the facet joint involvement and adjacent collection, computed tomography (CT)-guided aspiration and washout of the L4-L5 facet joint was performed during the second week of admission for diagnostic confirmation and source control. Culture of the aspirate again grew S. lugdunensis, confirming facet joint septic arthritis due to the same organism isolated from blood.

Differential diagnosis

The initial priority was to exclude compressive and infective spinal emergencies. Cauda equina syndrome was considered given urinary retention and saddle anaesthesia; however, MRI showed no compressive lesion, no significant canal stenosis, and no thecal sac compromise. Spinal epidural abscess was also considered in view of severe pain and elevated C-reactive protein, but MRI demonstrated no epidural collection. Post-procedural haematoma was less likely given the seven-week interval, the absence of haemorrhagic features on MRI, and subsequent microbiological confirmation of infection. Degenerative spinal disease could not account for the markedly elevated inflammatory markers or the imaging evidence of facet joint infection.

Overall, the combination of progressive post-procedural pain, focal MRI findings, and concordant microbiology from blood cultures and facet aspirate established the diagnosis of S. lugdunensisbacteraemia with bilateral L4-L5 facet joint septic arthritis and a small paraspinal collection.

Treatment

After blood cultures were obtained, empirical intravenous antimicrobials were commenced with vancomycin and cefepime to provide broad Gram-positive and Gram-negative coverage while awaiting culture identification and susceptibility results (dose adjustments were made according to renal function and therapeutic drug monitoring). Neurosurgical review did not recommend operative intervention because MRI showed no cauda equina compression, no epidural abscess, and no other surgical compressive lesion, and the patient was managed with targeted antimicrobial therapy plus image-guided source control.

Once S. lugdunensiswas confirmed and shown to be beta-lactam susceptible, treatment was streamlined to intravenous flucloxacillin (2 g every six hours, adjusted for renal function). A total of four weeks of intravenous therapy was completed from the first documented negative blood culture (hospital day six). CT-guided aspiration and washout of the affected L4-L5 facet joint was undertaken during the second week of admission to reduce local burden and provide microbiological confirmation.

On discharge, oral step-down therapy was used to complete a prolonged treatment course for bacteraemic deep spinal infection after clinical improvement, inflammatory marker response, microbiological clearance, and absence of a neurosurgical indication for operative management. Cephalexin was initially used as oral step-down therapy for one week. The patient was then started on clindamycin 300 mg every eight hours plus amoxicillin 500 mg every eight hours, with significant improvement in low back pain. At subsequent follow-up, clindamycin was discontinued and amoxicillin-clavulanate was continued every 12 hours for a further three to four weeks, with later extension for an additional two weeks pending interval MRI review.

The patient tolerated antimicrobial therapy without reported adverse effects and required no readmission or surgery. Overall antimicrobial duration comprised four weeks of intravenous therapy followed by prolonged oral step-down therapy, giving a total treatment course of approximately 12 weeks. Oral regimens were adjusted sequentially (cephalexin, then clindamycin plus amoxicillin, followed by amoxicillin-clavulanate) to complete therapy.

Outcome and follow-up

**Figure 2:**
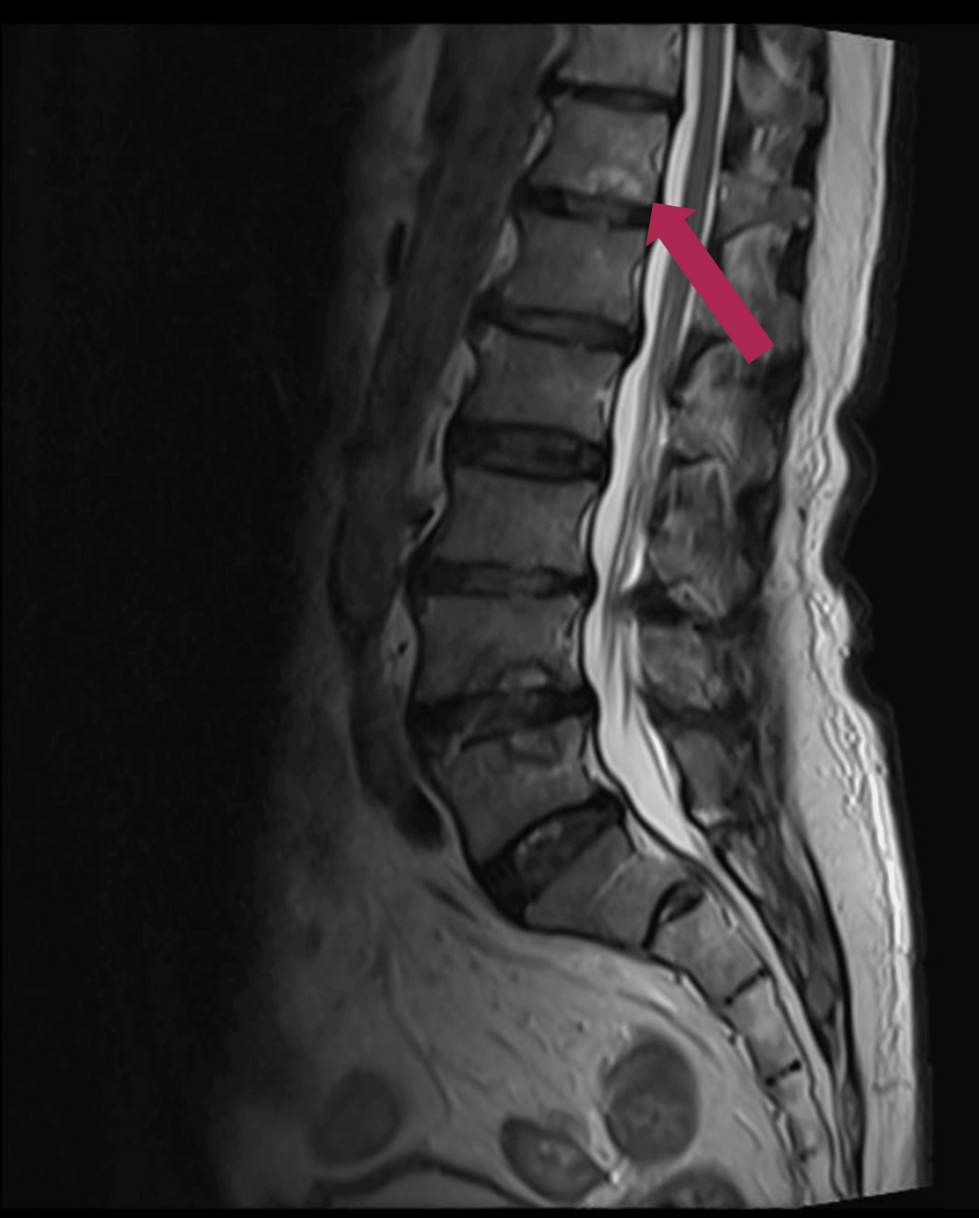
Follow-up lumbar spine MRI showing interval improvement of L4–L5 facet joint infection Follow-up MRI of the lumbar spine with and without contrast demonstrates interval improvement with mild residual inflammatory changes at the bilateral L4–L5 facet joints and no rim-enhancing fluid collection. No epidural collection or cauda equina compression is seen.

At follow-up one to two months after treatment initiation, the patient reported substantial improvement in back pain (approximately 80% reduction) and no recurrence of urinary symptoms or other neurological deficits. Inflammatory markers normalised during therapy (Table 4). Follow-up MRI demonstrated interval improvement in inflammatory changes at L4-L5 and reduction of the paraspinal collection, without new epidural collection or cauda equina compression (Figure 2). The patient tolerated antimicrobial therapy without reported adverse effects and required no readmission or surgical intervention.

**Table 4:** Follow-up laboratory results

Domain	Test / specimen	Result	Reference range / comment
Inflammatory markers	C-reactive protein (CRP)	1.60 mg/L	0–5 mg/L
	Procalcitonin	0.10 ng/mL	<0.50 ng/mL
	Erythrocyte sedimentation rate (ESR)	12 mm/hr	≈ 37 mm/hr
Haematology	White blood cell count (WBC)	5.02 × 10⁹/L	4.0–11.0 × 10⁹/L
	Haemoglobin	112 g/L	130–170 g/L
	Platelets	342 × 10⁹/L	150–400 × 10⁹/L

## Discussion

Staphylococcus lugdunensis virulence

This case demonstrates a rare but serious complication following lumbar radiofrequency ablation: bacteraemic bilateral facet joint septic arthritis caused by Staphylococcus lugdunensis, an organism whose virulence can resemble Staphylococcus aureus despite its classification as a coagulase-negative Staphylococcus [1,2,7]. Bloodstream isolation should therefore be treated as clinically significant rather than dismissed as contamination, with prompt antistaphylococcal therapy and evaluation for invasive foci [1-3,10]. Clumping factor production may also contribute to misidentification as S. aureusin routine workflows, reinforcing the need for careful microbiological interpretation and timely optimisation of therapy once susceptibilities are available [1,2]. Assessment for infective endocarditis remains important in S. lugdunensisbacteraemia, including in this case where echocardiographic evaluation showed no vegetation [3,10].

Radiofrequency ablation complications and procedural association

Lumbar radiofrequency ablation is an established treatment for appropriately selected patients with facet-mediated pain and is generally low risk [5,6]. However, progressive or refractory post-procedural pain, particularly with neurological or autonomic red flags, should prompt urgent evaluation for deep spinal infection even when fever and other systemic features are absent [7,9]. In this case, two mechanisms are plausible: direct inoculation during radiofrequency ablation or haematogenous seeding from an unrecognised distant focus [1,2,8]. The temporal relationship suggests a possible procedural association, but causality cannot be established from a single case report [1,2].

Facet joint septic arthritis and diagnostic challenge

Septic arthritis of the spinal facet joint is uncommon and may mimic degenerative disease or routine post-procedural discomfort [8,11]. MRI is the principal diagnostic modality for defining facet infection, detecting paraspinal or epidural extension, and excluding competing spinal emergencies [8,11]. In this patient, MRI demonstrated bilateral L4-L5 facet joint infection with a small paraspinal collection while excluding cauda equina compression and epidural abscess, which guided management and avoided unnecessary decompression [8,11]. The early re-presentation after the procedure with pain but no documented focal neurological deficit also illustrates how deep infection may evolve subtly over weeks and require reassessment when symptoms escalate [7,8].

Management principles

Management in this case followed core principles for invasive staphylococcal infection and deep spinal sepsis: early empirical broad-spectrum therapy, narrowing to targeted beta-lactam treatment after susceptibility results, microbiological clearance monitoring, and source control when feasible [1,4,8,9]. CT-guided aspiration and washout provided diagnostic confirmation and reduced local bacterial burden [8,9]. Clinical response was supported by serial inflammatory marker improvement, blood culture clearance, and follow-up MRI showing interval radiological improvement without new epidural collection [8].

Limitations and clinical relevance

As a single case, definitive causality between the procedure and infection cannot be established, and the source of bacteraemia remains uncertain. A longer follow-up would strengthen the assessment of recurrence risk and functional trajectory. Nevertheless, this case highlights the need to investigate progressive post-procedural pain with neurological red flags for deep spinal infection and to manage S. lugdunensisbacteraemia as a true pathogen requiring active evaluation and prolonged culture-directed therapy.

## Conclusions

Staphylococcus lugdunensis can cause invasive infection and should be treated as clinically significant when isolated from blood. This case of bacteraemic bilateral L4-L5 facet joint septic arthritis following lumbar radiofrequency ablation highlights that progressive or refractory post-procedural back pain, particularly with neurological or autonomic red flags, warrants urgent evaluation for deep spinal infection even in the absence of fever. Early MRI assessment, microbiological confirmation with documented blood culture clearance, source control when feasible, and prolonged culture-directed antistaphylococcal therapy can lead to sustained clinical and radiological improvement.
